# Bacterial isolate collection from switchgrass rhizosphere

**DOI:** 10.1128/mra.01133-23

**Published:** 2024-06-06

**Authors:** Keara L. Grady, Ashley Shade

**Affiliations:** 1 Great Lakes Bioenergy Research Center, Michigan State University, East Lansing, Michigan, USA; 2 Universite Claude Bernard Lyon 1, CNRS, INRAE, VetAgro Sup, Laboratoire d'Ecologie Microbienne LEM, CNRS UMR5557, INRAE UMR1418, Villeurbanne, France; University of Strathclyde, Glasgow, United Kingdom

**Keywords:** biofuel, rhizosphere, bacteria, plant microbiome, bioenergy, feedstock, perennial

## Abstract

We provide a collection of 78 bacterial isolates from the rhizosphere of switchgrass (*Panicum virgatum L*.) at the Lux Arbor Reserve in Delton, MI, a site of the Great Lakes Bioenergy Research Center (GLBRC), Michigan State University, MI, USA. We include information on isolation conditions and full-length 16S rRNA sequences.

## ANNOUNCEMENT

Switchgrass (*Panicum virgatum* L.) is a perennial grass and biofuel feedstock that offers economic and environmental benefits, including carbon sequestration in deep-rooting systems and growth on marginal lands with the need for minimal fertilization. Switchgrass harbors a relatively stable root-associated microbial community structure, especially compared to its more dynamic phyllosphere community (1–3). Thus, perennial crop microbiomes may offer insights into durable plant-microbe relationships.

Bacterial strains were isolated from the switchgrass rhizosphere (cultivar Cave-in-rock) at the Lux Arbor Reserve in Delton, MI, USA (42.4764, –85.4519). Roots were collected on 30 August 2018 from mature, pre-senescent plants using a 2.5 cm diameter, 20 cm depth soil corer to collect soils and roots at the base. In a biosafety cabinet, bulk soil was removed from ~2 g of root tissue per plant, then roots were sonicated in 10 mL of sterile phosphate-buffered saline +0.01% Tween-20 (PBS-T) for 2 min and incubated at 4°C for 1 h to settle large soil particles. Cells were pelleted from the supernatant at 6,000 × *g* for 10 min at 4°C. The pellet was resuspended in 1 mL PBS + 25% glycerol, flash-frozen in liquid nitrogen, and stored at −80°C.

Slurries were thawed on ice, mixed thoroughly, and serially diluted into sterile PBS-T. Aliquots of diluted slurry were spread onto either rhizosphere isolation medium (RIM) (4) or Reasoner’s 2A (R2A) agar + 0.5% succinate, containing 0.2 g/mL cycloheximide (cyc) to prevent fungal growth. Plates were incubated at 27°C in aerobic conditions for 24–72 h. Individual colonies were picked onto new RIM-cyc or R2A-cyc plates and purified by streaking for isolation, then stored in R2A + 25% glycerol at −80°C. Table 1 includes growth conditions for each strain.

**TABLE 1:** NCBI BLAST taxonomy, isolation media, and DNA conditions of 78 bacterial isolates from switchgrass rhizosphere, as described in this study

Isolate_ID	Assembly_ BLAST_Genus	Assemby_ BLAST_species	BLAST _Grade^*a*^	BLAST_Hit_ Accession	Isolation_ Media^*b*^	DNA_Method	NCBI_Accession_ number	SRA_Accession_ number
PvR001	*Rhodococcus*	*fascians*	99.7	NR_119126	RIM	Zymo Quick-DNA Fungal/Bacterial 96 kit	OR754732	SRR27348840
PvR006	*Streptomyces*	*zaomyceticus*	100	NR_112376	RIM	Zymo Quick-DNA Fungal/Bacterial miniprep kit	OR754733	SRR27348839
PvR008	*Paenibacillus*	*terrae*	99.1	NR_025170	RIM	Zymo Quick-DNA Fungal/Bacterial miniprep kit	OR754734	SRR27348828
PvR012	*Burkholderia*	*diffusa*	99.8	NR_042633	RIM	Zymo Quick-DNA Fungal/Bacterial 96 kit	OR754735	SRR27348817
PvR013	*Chryseobacterium*	*lactis*	99.7	NR_126256	RIM	CTAB-phenol-chloroform with acetone freeze/thaw cycle	OR754736	SRR27348806
PvR015	*Chryseobacterium*	*lactis*	99.1	NR_126256	RIM	CTAB-phenol-chloroform with acetone freeze/thaw cycle	OR754737	SRR27348785
PvR016	*Rhizobium*	*phaseoli*	99.3	NR_113671	RIM	Zymo Quick-DNA Fungal/Bacterial 96 kit	OR754738	SRR27348774
PvR017	*Burkholderia*	*ambifaria*	99.8	NR_074687	RIM	Zymo Quick-DNA Fungal/Bacterial 96 kit	OR754739	SRR27348763
PvR018	*Paenibacillus*	sp.	99.8	NR_112906	RIM	CTAB-phenol-chloroform with acetone freeze/thaw cycle	OR754740	SRR27348796
PvR019	*Mycolicibacterium*	*mucogenicum*	99.6	NR_042919	RIM	Zymo Quick-DNA Fungal/Bacterial miniprep kit	OR754741	SRR27348795
PvR021	*Pantoea*	*agglomerans*	99.9	NR_111998	RIM	Zymo Quick-DNA Fungal/Bacterial 96 kit	OR754742	SRR27348838
PvR022	*Burkholderia*	*contaminans*	99.9	NR_104978	RIM	Zymo Quick-DNA Fungal/Bacterial 96 kit	OR754743	SRR27348837
PvR023	*Burkholderia*	*ambifaria*	99.7	NR_074687	RIM	Zymo Quick-DNA Fungal/Bacterial 96 kit	OR754744	SRR27348836
PvR024	*Cupriavidus*	*necator*	99.9	NR_102851	RIM	Zymo Quick-DNA Fungal/Bacterial 96 kit	OR754745	SRR27348835
PvR025	*Agrobacterium*	*rhizogenes*	99.9	NR_113607	RIM	Zymo Quick-DNA Fungal/Bacterial 96 kit	OR754746	SRR27348834
PvR029	*Novosphingobium*	*lindaniclasticum*	99.5	NR_118312	RIM	Zymo Quick-DNA Fungal/Bacterial 96 kit	OR754747	SRR27348833
PvR033	*Burkholderia*	*contaminans*	99.6	NR_104978	RIM	Zymo Quick-DNA Fungal/Bacterial 96 kit	OR754748	SRR27348832
PvR034	*Streptomyces*	*spiroverticillatus*	99.6	NR_112582	RIM	CTAB-phenol-chloroform with acetone freeze/thaw cycle	OR754749	SRR27348831
PvR038	*Burkholderia*	*stabilis*	99.9	NR_114522	RIM	Zymo Quick-DNA Fungal/Bacterial 96 kit	OR754750	SRR27348830
PvR040	*Rhodococcus*	*qingshengii*	99.8	NR_115708	RIM	Zymo Quick-DNA Fungal/Bacterial 96 kit	OR754751	SRR27348829
PvR041	*Enterobacter*	*ludwigii*	100	NR_042349	RIM	Zymo Quick-DNA Fungal/Bacterial 96 kit	OR754752	SRR27348827
PvR042	*Pseudomonas*	*protegens*	99.9	NR_114749	RIM	Zymo Quick-DNA Fungal/Bacterial 96 kit	OR754753	SRR27348826
PvR043	*Novosphingobium*	*capsulatum*	99.7	NR_113591	RIM	CTAB-phenol-chloroform with acetone freeze/thaw cycle	OR754754	SRR27348825
PvR044	*Rhodococcus*	*oryzae*	99.8	NR_170410	RIM	Zymo Quick-DNA Fungal/Bacterial miniprep kit	OR754755	SRR27348824
PvR045	*Novosphingobium*	*lindaniclasticum*	99.3	NR_118312	RIM	Zymo Quick-DNA Fungal/Bacterial 96 kit	OR754756	SRR27348823
PvR048	*Burkholderia*	*stabilis*	99.8	NR_114522	RIM	CTAB-phenol-chloroform with acetone freeze/thaw cycle	OR754757	SRR27348822
PvR052	*Paenibacillus*	sp.	99.8	NR_112906	RIM	Zymo Quick-DNA Fungal/Bacterial 96 kit	OR754758	SRR27348821
PvR053	*Paenibacillus*	sp.	99.9	NR_112906	RIM	CTAB-phenol-chloroform with acetone freeze/thaw cycle	OR754759	SRR27348820
PvR055	*Chryseobacterium*	*lactis*	99	NR_126256	RIM	Zymo Quick-DNA Fungal/Bacterial miniprep kit	OR754760	SRR27348819
PvR056	*Dyella*	*japonica*	100	NR_114075	RIM	Zymo Quick-DNA Fungal/Bacterial miniprep kit	OR754761	SRR27348818
PvR057	*Agromyces*	*allii*	99.7	NR_043931	RIM	Zymo Quick-DNA Fungal/Bacterial miniprep kit	OR754762	SRR27348816
PvR058	*Nocardioides*	*endophyticus*	99.7	NR_135731	RIM	Zymo Quick-DNA Fungal/Bacterial miniprep kit	OR754763	SRR27348815
PvR059	*Nocardioides*	*endophyticus*	99.7	NR_135731	RIM	Zymo Quick-DNA Fungal/Bacterial 96 kit	OR754764	SRR27348814
PvR060	*Novosphingobium*	*hassiacum*	98.7	NR_028962	RIM	Zymo Quick-DNA Fungal/Bacterial miniprep kit	OR754765	SRR27348813
PvR061	*Enterobacter*	*ludwigii*	100	NR_042349	RIM	Zymo Quick-DNA Fungal/Bacterial 96 kit	OR754766	SRR27348812
PvR062	*Mycobacterium*	*hackensackense*	99.4	NR_115184	RIM	CTAB-phenol-chloroform with acetone freeze/thaw cycle	OR754767	SRR27348811
PvR063	*Burkholderia*	*stabilis*	99.7	NR_114522	RIM	Zymo Quick-DNA Fungal/Bacterial 96 kit	OR754768	SRR27348810
PvR064	*Sphingobium*	*chlorophenolicum*	99.5	NR_113840	RIM	Zymo Quick-DNA Fungal/Bacterial miniprep kit	OR754769	SRR27348809
PvR065	*Mesorhizobium*	*jarvisii*	99.9	NR_135858	RIM	Zymo Quick-DNA Fungal/Bacterial 96 kit	OR754770	SRR27348808
PvR066	*Amycolatopsis*	*vastitatis*	99.5	NR_164904	RIM	CTAB-phenol-chloroform with acetone freeze/thaw cycle	OR754771	SRR27348807
PvR072	*Rhizobium*	*alamii*	99.9	NR_042687	RIM	Zymo Quick-DNA Fungal/Bacterial 96 kit	OR754772	SRR27348805
PvR077	*Burkholderia*	*contaminans*	99.8	NR_104978	RIM	Zymo Quick-DNA Fungal/Bacterial 96 kit	OR754773	SRR27348794
PvR079	*Dyella*	*koreensis*	99.6	NR_113947	RIM	Zymo Quick-DNA Fungal/Bacterial miniprep kit	OR754774	SRR27348793
PvR083	*Pseudomonas*	*frederiksbergensis*	99.8	NR_117177	RIM	Zymo Quick-DNA Fungal/Bacterial 96 kit	OR754775	SRR27348792
PvR085	*Novosphingobium*	*capsulatum*	99.7	NR_113591	RIM	Zymo Quick-DNA Fungal/Bacterial 96 kit	OR754776	SRR27348791
PvR087	*Dyella*	*Koreensis*	99.1	NR_113947	RIM	Zymo Quick-DNA Fungal/Bacterial 96 kit	OR754777	SRR27348790
PvR089	*Dyella*	*koreensis*	99.2	NR_113947	RIM	Zymo Quick-DNA Fungal/Bacterial miniprep kit	OR754778	SRR27348789
PvR090	*Novosphingobium*	*capsulatum*	99.8	NR_113591	RIM	Zymo Quick-DNA Fungal/Bacterial miniprep kit	OR754779	SRR27348788
PvR092	*Lysobacter*	*soli*	99.3	NR_116074	RIM	Zymo Quick-DNA Fungal/Bacterial 96 kit	OR754780	SRR27348787
PvR093	*Mesorhizobium*	*amorphae*	99.9	NR_114122	RIM	CTAB-phenol-chloroform with acetone freeze/thaw cycle	OR754781	SRR27348786
PvR094	*Mycolicibacterium*	*septicum*	99.9	NR_114492	RIM	Zymo Quick-DNA Fungal/Bacterial 96 kit	OR754782	SRR27348784
PvR095	*Novosphingobium*	*capsulatum*	99.7	NR_113591	RIM	Zymo Quick-DNA Fungal/Bacterial 96 kit	OR754783	SRR27348783
PvR096	*Mycolicibacterium*	*mucogenicum*	99.4	NR_114656	RIM	Zymo Quick-DNA Fungal/Bacterial 96 kit	OR754784	SRR27348782
PvR097	*Microbacterium*	*flavescens*	99.5	NR_029350	RIM	Zymo Quick-DNA Fungal/Bacterial 96 kit	OR754785	SRR27348781
PvR098	*Paenibacillus*	sp.	99.6	NR_112906	RIM	Zymo Quick-DNA Fungal/Bacterial 96 kit	OR754786	SRR27348780
PvR100	*Nocardioides*	*cavernae*	99.7	NR_156135	RIM	Zymo Quick-DNA Fungal/Bacterial miniprep kit	OR754787	SRR27348779
PvR101	*Sphingobium*	*indicum*	99.3	NR_102886	RIM	Zymo Quick-DNA Fungal/Bacterial 96 kit	OR754788	SRR27348778
PvR102	*Dyella*	*marensis*	100	NR_042691	RIM	CTAB-phenol-chloroform with acetone freeze/thaw cycle	OR754789	SRR27348777
PvR103	*Polaromonas*	*eurypsychrophila*	99.2	NR_149767	RIM	Zymo Quick-DNA Fungal/Bacterial 96 kit	OR754790	SRR27348776
PvR104	*Mycolicibacterium*	*neworleansense*	99.5	NR_115113	RIM	Zymo Quick-DNA Fungal/Bacterial 96 kit	OR754791	SRR27348775
PvR105	*Mesorhizobium*	*jarvisii*	100	NR_135858	RIM	Zymo Quick-DNA Fungal/Bacterial miniprep kit	OR754792	SRR27348773
PvR106	*Rhizobium*	*tibeticum*	99.1	NR_116254	RIM	CTAB-phenol-chloroform with acetone freeze/thaw cycle	OR754793	SRR27348772
PvR107	*Methylobacterium*	*phyllostachyos*	99.9	NR_108242	RIM	Zymo Quick-DNA Fungal/Bacterial miniprep kit	OR754794	SRR27348771
PvR108	*Chryseobacterium*	*lactis*	99.4	NR_126256	RIM	Zymo Quick-DNA Fungal/Bacterial 96 kit	OR754795	SRR27348770
PvR112	*Sphingobacterium*	*cladoniae*	99.4	NR_108441	RIM	Zymo Quick-DNA Fungal/Bacterial 96 kit	OR754796	SRR27348769
PvR114	*Burkholderia*	*ambifaria*	99.9	NR_074687	RIM	Zymo Quick-DNA Fungal/Bacterial 96 kit	OR754797	SRR27348768
PvR115	*Agrobacterium*	*rhizogenes*	99.9	NR_113607	RIM	Zymo Quick-DNA Fungal/Bacterial 96 kit	OR754798	SRR27348767
PvR116	*Lelliottia*	*nimipressuralis*	99.5	NR_044976	RIM	Zymo Quick-DNA Fungal/Bacterial 96 kit	OR754799	SRR27348766
PvR117	*Rhizobium*	*alamii*	99.9	NR_042687	R2A	Zymo Quick-DNA Fungal/Bacterial 96 kit	OR754800	SRR27348765
PvR118	*Pseudomonas*	*protegens*	99.8	NR_114749	R2A	Zymo Quick-DNA Fungal/Bacterial 96 kit	OR754801	SRR27348764
PvR119	*Pseudomonas*	*protegens*	99.8	NR_114749	R2A	Zymo Quick-DNA Fungal/Bacterial 96 kit	OR754802	SRR27348804
PvR122	*Roseococcus*	*suduntuyensis*	98.7	NR_044369	R2A	Zymo Quick-DNA Fungal/Bacterial miniprep kit	OR754803	SRR27348803
PvR126	*Burkholderia*	*ambifaria*	99.7	NR_074687	R2A	Zymo Quick-DNA Fungal/Bacterial 96 kit	OR754804	SRR27348802
PvR127	*Burkholderia*	*ambifaria*	99.7	NR_074687	R2A	Zymo Quick-DNA Fungal/Bacterial 96 kit	OR754805	SRR27348801
PvR132	*Burkholderia*	*stabilis*	99.8	NR_114522	R2A	Zymo Quick-DNA Fungal/Bacterial 96 kit	OR754806	SRR27348800
PvR133	*Paenibacillus*	*peoriae*	99.9	NR_117743	R2A	Zymo Quick-DNA Fungal/Bacterial miniprep kit	OR754807	SRR27348799
PvR147	*Leclercia*	sp.	97.5	NR_114154	R2A	Zymo Quick-DNA Fungal/Bacterial 96 kit	OR754808	SRR27348798
PvR148	*Paenibacillus*	sp.	99.8	NR_112906	R2A	Zymo Quick-DNA Fungal/Bacterial miniprep kit	OR754809	SRR27348797

^*a*^ The BLAST grade, an informative alternative to percent sequence identity, is a weighted score composed of the *e*-value, pairwise identity, and query coverage reported for the top hit.

^*b*^ RIM is rhizosphere isolation media and R2A is Reasoner’s 2A media.

**Fig 1:**
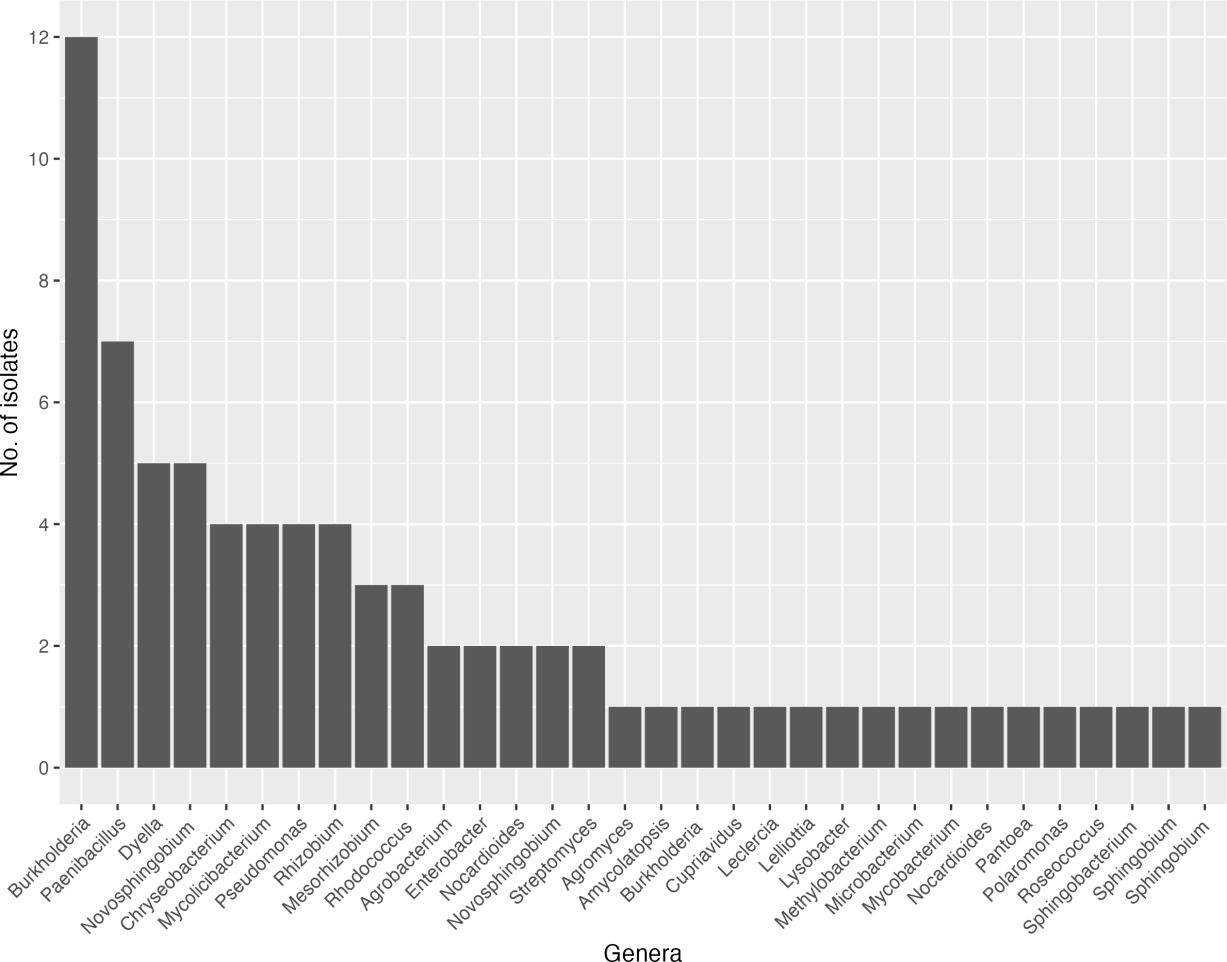
Taxonomic summary of the bacterial collection cultivated from switchgrass rhizosphere, genera identified by full-length 16S rRNA gene sequencing (Sanger) with taxonomy assigned by match (BLAST) to the NCBI 16S rRNA database, determined using Geneious Prime Megablast function to access the NCBI 16S ribosomal RNA database using default parameters on 18 January 2023

Genomic DNA (gDNA) was extracted using a Quick DNA fungal-bacterial miniprep kit (Zymo Research #D6005). For some isolates, gDNA was extracted using an acetone freeze-thaw cycle and enzymatic lysis followed by a liquid extraction (5, 6). Full-length 16S rRNA sequences were amplified using the 27F-1492R primer set (5′-AGAGTTTGATCCTGGCTCAG-3′ and 5′-GGTTACCTTGTTACGACTT-3′) (7) and Phusion High-fidelity polymerase (New England Biolabs) under the following conditions: denaturation at 98°C for 1 min, then 25 cycles of denaturation (98°C for 10 s), annealing (58°C for 20 s), and extension (72°C for 45 s). Amplicons were cleaned using a Wizard-SV gel/PCR Clean-up kit (Promega) and Sanger-sequenced on the ABI 3730xl at the MSU Research Technology Support Facility (rtsf.natsci.msu.edu). Amplicons were sequenced from both directions using the above primers, and were analyzed using Geneious Prime software v 2021.2.2 (Dotmatics, Table 1; Fig. 1).

## Data Availability

Full-length 16S consensus sequences are available at NCBI, accession OR754732-OR754809 and raw reads are available at the NCBI SRA under Bioproject PRJNA1056287, accession numbers SRR27348763-SRR27348840. The bacterial collection and individual isolates are available by email request to The Great Lakes Bioenergy Research Center at Michigan State University, USA (glbrc@msu.edu).
